# Biomass power plant prospects in Indonesia’s energy transition: IPP and PLN perspectives

**DOI:** 10.1016/j.heliyon.2024.e38970

**Published:** 2024-10-05

**Authors:** Indra A. Aditya, Hendry Timotiyas Paradongan, Iswan Prahastono, Sudjono Kosasih, Kevin M. Banjar-Nahor, Ngapuli Irmea Sinisuka

**Affiliations:** a PLN Research Institute, PT PLN (Persero), Jakarta, 12760, Indonesia; b School of Business and Management, Bandung Institute of Technology, Bandung, 40132, Indonesia; c School of Electrical Engineering and Informatics, Bandung Institute of Technology, Bandung, 40132, Indonesia; d Prima Gasifikasi Indonesia, PT Prima Gasifikasi Indonesia, Tanjung Batu, 29662, Indonesia

**Keywords:** Renewable energy, Renewable energy tariffs, Energy transition, Biomass, Indonesia, De-dieselization

## Abstract

In an attempt to accelerate the energy transition, the government of Indonesia released a new regulation regarding renewable energy tariffs, including for biomass power plants (BPPs). However, a gap persists in the academic literature focusing on biomass power plants under these new tariffs. This study examines the role of biomass power plants (BPP) in meeting Indonesia's renewable energy targets, with a specific focus on recently introduced renewable energy tariffs. Three scenarios were developed to comprehensively understand Indonesia's state-owned electricity company (PLN) perspective and Independent Power Producers (IPPs) perspective, with a case study in Kundur Island, Riau Province. The financial analysis for IPPs shows in an NPV of $1.8 million under the initial tariff, while the expansion tariff results in an NPV of -$0.3 million. Sensitivity analysis reveals that a capacity factor exceeding 94.5 % is required to yield a positive NPV under the expansion tariff. From PLN's perspective, BPPs under the initial tariff correspond to an NPV of $8.48 million, and a B-C ratio of 2.31, resulting in a financially viable project, due to substantial cost savings from de-dieselization. Furthermore, the emission analysis highlights an 83.7 % decrease in emissions emitted with the utilization of BPPs compared to diesel power plants. These findings underscore the need to incorporate average generation cost in formulating renewable energy tariffs. Ensuring renewable energy projects are economically sustainable for both PLN and IPPs, stimulates widespread adoption and sustains cost-savings, all while advancing energy transition and making energy more accessible for everyone.

## 1 Introduction

In recent decades, the global energy landscape has undergone a transformative shift, with increasing attention directed towards sustainable and renewable energy sources. Nations worldwide are prioritizing their energy security and the repercussions of climate change. This has led to a concentrated effort to explore economically viable and ecologically sustainable with socially and environmentally sustainable alternative energy sources. To ensure the fulfillment of future electricity consumption with the repercussions of climate change and sustainability, the Indonesian government is committing to a 23 % renewable energy mix by the end of 2025 [[1], [2], [3]] and attain net-zero emission by 2060 [4,5]. However, by the end of 2021, the country's renewable energy mix remains at 11.5 % of its energy mix [6]. By the end of 2025, Indonesia needs to increase its renewable energy mix by 11.5 % to attain the 23 % renewable energy mix target.

Energy plays a pivotal role in Indonesia's economic growth, where the attainment of sustainable development of the energy sector holds indispensable significance for the overall progress of the nation's development [7]. From 1990 to 2020, the country's electricity consumption has grown from 29.48 TWh to 268.12 TWh or more than 800 % [8]. According to Indonesia's Minister of Energy and Mineral Resources, Indonesia's oil reserves are only sufficient for 9.5 years [9]. To maintain energy security, the government is actively adding oil and gas exploration activities and encouraging energy transition in its power sector. In support of this energy transition, PLN introduced the de-dieselization program aimed at reducing diesel-based power plants usage. The country's biomass potential is largely unutilized, according to Refs. [[10], [11], [12]], Indonesia has around 32 GWe of biomass potential, equivalent to 71 % of PLN's installed capacity. However, the utilization of biomass for electricity generation is still lacking, with an installed capacity of around 157.42 MW [13], or 0.005 % utilization from its full potential.

Several studies have researched biomass power plants. Pan et al. [14] conducted a feasibility analysis on various distributed energy systems in Chongming using RETScreen software, according to Pan's research, the introduction of a biomass system is essential for covering the energy demand and minimizing energy import, highlighting the importance of biomass power plants in providing reliable energy sources. In another study by Prasad and Aturi [15] on the prospects of sustainable biomass-based power generation in Fiji, determined that a 10 MW biomass power plant would require approximately 60,000 tonnes of biomass. This demand could be supplied from the forest residue from logging in the western division of Viti Levu, highlighting the importance of biomass systems to reduce waste, while generating electricity. Delivand et al. [16] conducted an economic feasibility assessment on 5 projected biomass power plants in Thailand, with a corresponding capacity of 5, 8, 10, 15, and 20 MW. Based on the research, the most influential parameters affecting the project profitability are capacity factor, followed by electricity tariffs, and fuel price. Chambon et al. [17] conducted a techno-economic assessment of biomass gasification, where it concluded that biomass gasification systems offered a greater resilience to operational disruptions, and a more reliable supply of power with low LCOE. Cardoso et al. [18] conducted a feasibility analysis of an 11 MW gasification biomass gasification power plant dealing with forestry residues blends for electricity production in Portugal, according to the research, the project is considered feasible with an NPV of 2.367 million Euro. Despite the viability of the project, the economic performance is strongly reliant on revenues from electricity sales regulated by uncertain tariffs and reimbursement. The research also concluded that biomass power plant with gasification technology has an advantage in terms of greenhouse gas emission control.

The Indonesian government released a new renewable energy tariff regulation [19] to accelerate the energy transition, serving as a benchmark for PLN's power purchase agreement (PPA) price. The tariffs are categorized into initial and expansion categories, with the expansion tariffs being lower than the initial tariffs. In a study conducted by Ref. [20] on a photovoltaic (PV) power plant in Nias based on the new tariffs, it concluded that other incentives are needed to make the facility financially viable. However, a research gap exists in the academic literature focusing on biomass power plants under these new tariffs. Additionally, there is insufficient research on the utilization and role of biomass power plants in the energy transition. This study also seeks to fill the gap in understanding the feasibility of biomass power from both Independent Power Producer (IPP) and Perusahaan Listrik Negara (PLN) perspectives, offering comprehensive insights for all stakeholders in the energy sector.

## 2 Literature review

### 2.1 Biomass power plant tariffs

The Indonesian government recently introduced a new renewable energy tariff regulation [19], regulating tariff benchmarks for various renewable energy-based electricity in Indonesia, including biomass power plants. The tariff calculation took into account several factors, such as the facility's location, capacity, and years of operation. The tariffs were also categorized into two types, initial tariffs, and expansion tariffs.

The power plant location in this study is located in Kundur Island, Riau Islands Province, Indonesia. A summary of the biomass power plant initial tariffs for this study is shown in Table 1 and the summary of biomass power plant expansion tariffs is shown in Table 2.

**Table 1:** Biomass power plant tariffs - initial [19].

**Capacity (MW)**	**BPP Tariff for Year 1**–**10 ($/kWh)**	**BPP Tariff for Year 11**–**25 ($/kWh)**
≤ **1**	0.1386	0.0924
> **1**–**3**	0.1288	0.0859
> **3**–**5**	0.1224	0.0816
> **5**–**10**	0.1183	0.0789
> **10**–**20**	0.1114	0.0743

**Table 2:** Biomass power plant tariffs - expansion [19].

**Capacity (MW)**	**BPP Tariff for Year 1**–**10 ($/kWh)**	**BPP Tariff for Year 11**–**25 ($/kWh)**
≤ **1**	$0.1109	$0.07
> **1**–**3**	$0.1030	$0.0687
> **3**–**5**	$0.0979	$0.0653
> **5**–**10**	$0.0947	$0.0631
> **10**–**20**	$0.0892	$0.0594

Based on the tables above, the tariff structure is designed to incentivize small-scale projects in the biomass sector, as the structures for large-scale facilities are considerably inferior to those for small-scale facilities. Prahastono et al. [21] conducted an assessment of the Nusa Penida photovoltaic power plant based on Indonesia's photovoltaic tariffs, according to the research results, the newly implemented tariffs are inadequate to make the project financially viable for IPPs. Unfortunately, according to the author's knowledge, research focusing on the viability of the new renewable energy tariffs for biomass power plants is still absent. Therefore, this study aims to fill the significant gap by evaluating the financial viability of a biomass power plant utilizing the newly implemented tariffs.

Three scenarios were developed to assess the project's viability based on various perspectives. The first scenario, referred to as the IPP scenario was created to assess the viability of the biomass power plant project based on the IPP perspective, utilizing the initial biomass tariffs. The second scenario, referred to as the IPP expansion scenario was created to assess the viability of the biomass power plant project based on the IPP perspective, utilizing the expansion biomass power plant tariffs. The third scenario, the PLN scenario was created to assess the viability of biomass power plants from the perspective of Indonesia's state-owned electricity company (PLN). This scenario involves the integration of the biomass power plant into Kundur's power system to assess its viability, considering the associated cost-savings resulting from the process of de-dieselization.

### 2.2 De-dieselization program

**Fig. 1:**
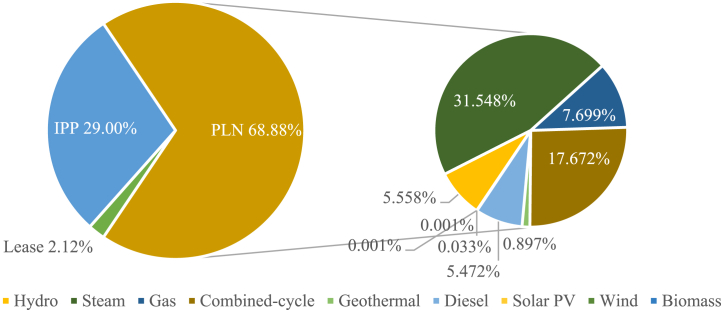
PLN's installed capacity.

Indonesia's power system is characterized by a diverse portfolio of power generation sources including diesel, coal, and natural gas, and a range of renewable energy sources including wind, hydro, solar, biomass, and geothermal. PLN, Indonesia's state-owned electricity company, plays a crucial role in planning, generating, transmitting, and distributing electricity across Indonesia. According to PLN's report [22], the company and its subsidiaries operate 6143 power plants, with an installed capacity of 44,467.75 MW, while IPPs contributed 18,722.32 MW and leased power plants contributed 1365.97 MW, resulting in a combined installed capacity of 64,553 MW. A breakdown of PLN's installed power capacity is shown in Fig. 1.

**Fig. 2:**
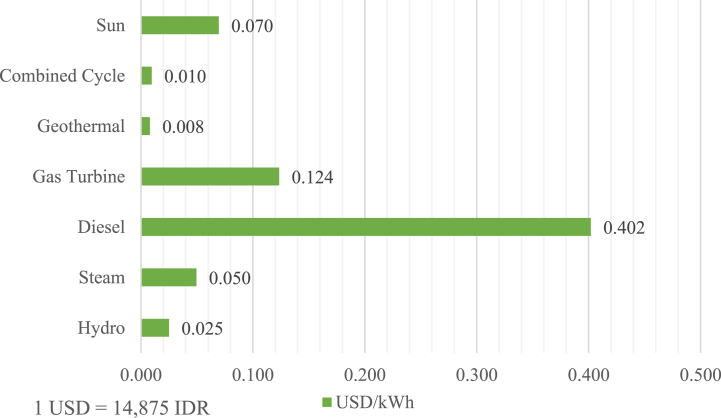
PLN's average generation cost [24].

The pricing structure of electricity provided by PLN is subject to government regulation, primarily aimed at ensuring the accessibility and affordability of electricity [23]. As a result, decreasing the generation cost is essential for maintaining PLN's financial sustainability. According to the PLN statistics report [24], the company's average generation cost is Rp.1473/kWh, equivalent to $0.1/kWh. Notably, the generation cost of diesel power plants averages $0.402/kWh, considerably higher compared to other technology, as shown in Fig. 2.

Furthermore, in the Riau Islands Province, PLN's selling tariff is averaging at 1272 IDR/kWh [22], equivalent to $0.085/kWh, considerably lower compared to PLN's average diesel-based generation cost. Based on this information, it can be concluded that the electricity generation cost associated with diesel power plants substantially surpasses the electricity selling price to customers, resulting in financial losses for every electricity provided by diesel power plants. To accelerate the energy transition, and address these financial challenges, PLN initiated the de-dieselization program. The program aimed at converting diesel-based facilities into renewable alternatives.

Kundur Island's power system is predominantly powered by diesel-based power plants, as shown in Table 3. As part of the de-dieselization program, PLN is arranging to expand the Kundur 900 kW Biomass Power Plant by 2.4 MW [25]. It's important to mention that the biomass power plant is operated by an IPP, and consequently, the new PPA tariff will be based on the new renewable energy tariff regulation. It is also important to mention, that Kundur power system is also supplied by diesel power plants, from which it also obtains electricity. Based on those aspects, Kundur Biomass Power Plant appears as a suitable case study for this research.

**Table 3:** Power plants in Kundur power system.

Power Plant Name	Fuel Type	Ownership	Installed Capacity	Generation Capacity
**Tanjung Batu Diesel Power Plant**	Diesel	PLN	2.4 MW	1.68 MW
**Tanjung Batu Lease Power Plant**	Diesel	Lease	4 MW	4 MW
**Kundur Diesel Power Plant**	Diesel	PLN	7.62 MW	5.75 MW
**Tanjung Batu IPP Biomass Power Plant**	Biomass	IPP	0.9 MW	0.9 MW
**Total Capacity**			14.92 MW	12.33 MW

**Fig. 3:**
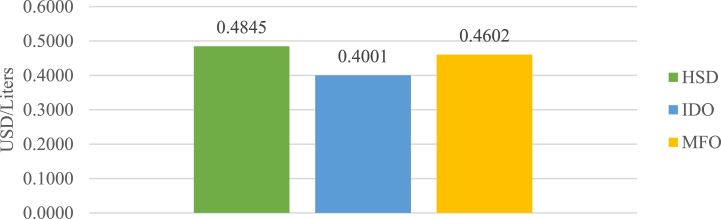
PLN's average fuel cost [22].

To conduct a thorough evaluation of the project's viability, this study considers both the IPP perspective and the PLN perspective. In assessing the project's viability based on the PLN perspective, referred to as the PLN scenario, this study utilizes the new renewable energy tariffs as the electricity purchase price for PLN. Additionally, it takes into account the cost savings accruing to PLN from the reduction of diesel power plant usage. Fig. 3 illustrates a summary of PLN's expenses related to fuel for diesel power plants, divided into High-Speed Diesel (HSD), Industrial Diesel Oil (IDO), and Marine Fuel Oil (MFO).

### 2.3 Biomass power plant

Indonesia presents a promising opportunity for biomass technology due to its extensive potential of biomass resources [26]. This potential is particularly significant given the technology's advantages in emission reduction [27], and its widespread availability. Harnessing biomass effectively through the gasification process represents a viable strategy for utilizing these resources [28]. For instance, the Kundur power plant on Kundur Island utilizes gasification technology to convert biomass into synthetic gas (syngas). The gasification process improves the efficiency of energy conversion, as demonstrated in a study by Huang et al. [29]. The gasification process involves the thermal-chemical conversion of biomass into syngas through partial oxidation under controlled oxygen condition [30]. The resulting syngas predominantly comprises hydrogen, carbon monoxide, methane, higher hydrocarbons, and, if air is used, nitrogen [31,32]. Gasification technology is particularly advantageous for distributed power generation systems, making it suitable for applications involving widely dispersed resources with relatively low energy density [33,34]. This process involves the transformation of solid or liquid organic materials into both a gas-vapor phase and a residual solid [35]. A study by Paletto et al. [36] highlights the importance of a short feedstock supply chain in reducing the environmental impact of biomass energy facilities. The research particularly emphasizes the benefits of optimizing the use of forest woodchips through processes such as debarking and drying to enhance environmental sustainability. Given the substantial prospective of biomass resources, the depletion of fossil fuel, and the government's commitment to the energy transition, biomass power plants emerge as a suitable prospect for the country's future energy landscape.

**Fig. 4:**
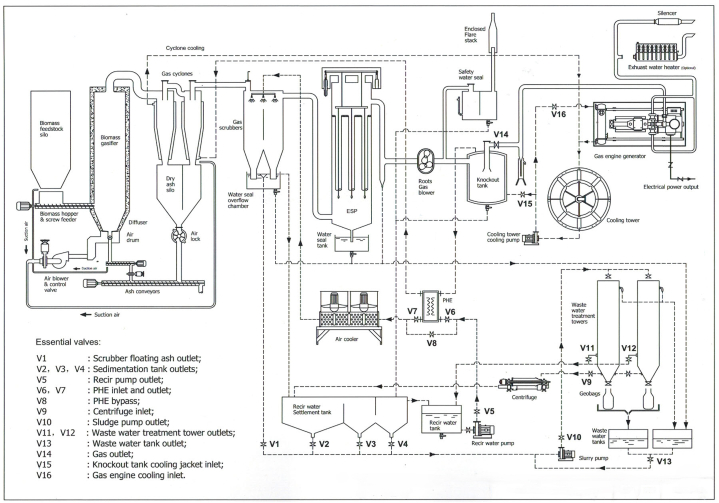
Configuration of Kundur biomass power plant [41].

The process of electricity generation using biomass with gasification technology, can be divided into two, syngas production process and electricity production process. The syngas production process converts the biomass into biogas, which then will be used in the electricity production process. As shown in Fig. 4, the process begins with the transport of harvested biomass to a designated storage site, commonly referred to as a silo. Subsequently, the biomass undergoes systematic introduction into the gasification system through mechanisms like the biomass hopper and screw feeder, ensuring a consistent and controlled feed rate. This process gains momentum with the controlled induction of air into an air drum, facilitated by air blowers and a regulating valve. The air is then injected into the gasifier chamber through a diffuser, maintaining controlled combustion parameters [33,37] and catalyzing the gasification process [34,38]. It is also important to mention, that this biomass power plant employs a bubbling fluidized-bed type of gasifier, offering faster reaction rates, and the ability to be constructed in larger sizes [39,40].

The gas output emerges from the upper reaches of the gasifier and advances to a cyclone unit. Here, the gas stream undergoes a centrifugal separation mechanism, effectively eliminating particulate contaminants such as char particles, ash, and some higher hydrocarbons or tar [31] that could jeopardize further process [42]. The gas that emerges from this stage is notably purified, setting the stage for the next pivotal phase. The purified gas then proceeds to a stage where heated air is extracted from the cyclone's cooling layers. This stream of hot air can be repurposed for various auxiliary applications, such as heating and cooling [43], and bolstering the overall energy efficiency of the system [44]. Concurrently, the separated ash descends and is collected within an ash silo, ensuring responsible disposal or potential utilization.

The next step is conducted to increase the purity of the produced gas stream, aiming to not only lower the gas stream's temperature but also to remove residual impurities and contaminants. As the gas moves through the scrubber, droplets of water are dispersed, precipitating into an overflow chamber and subsequently collected within a designated reservoir. The result of this exacting treatment is the emergence of a cooled and significantly purified gas stream, ready for further refinement. The treated gas proceeds to an Electrostatic Precipitator (ESP), a particle control device that uses electrical forces to move particles out of the following gas stream and onto a collector area [45]. The gas enters the base of the ESP and moves through electrode chambers. These chambers impart an electrostatic charge to suspended particles in the gas stream [46], such as contaminants like tar [38]. This charge prompts the particles to migrate towards and adhere to the electrode surfaces, segregating impurities from the gas stream. Emerging from the ESP, the gas is pure which is then extracted by a Roots Gas Blower, designed for consistent and controlled effluent flow. Simultaneously, byproducts from this purification process, including entrained water droplets and accumulated particulate matter, are directed into an ESP effluent reservoir for subsequent disposal or appropriate treatment. The process begins by directing the purified gas stream into a Roots Gas Blower, maintaining a carefully predefined velocity. The gas stream advances to a knockout tank for a key separation process. Within this chamber, the gas undergoes a separation mechanism, effectively disentangling it from any residual moisture or water content that might persist [47]. The purified gas or syngas then proceed as an energy resource for the gas engine to produce electricity.

## 3 Methodology

To achieve the research objective, this study utilizes RETScreen software, which has proven to be capable of assessing the feasibility of various renewable-energy projects, including biomass [14,15]. To assess the viability of the 2.4 MW biomass power plant based on PLN and IPPs perspectives, this study is divided into two parts. The first part focuses on the feasibility study of a 2.4 MW biomass power plant project to examine the role of the newly implemented renewable energy tariffs, viewed from IPP perspectives. The second part focuses on the calculation of electricity production cost provided by diesel power plants to determine the feasibility of a 2.4 MW biomass power plant project based on the PLN perspective.

### 3.1 Climate data

The location of the biomass power plant in this study is Kundur Island, Riau Islands Province, Indonesia, the climate data was gathered using RETScreen. The variations of climate data used in the study's calculation are shown in Table 4.

**Table 4:** Kundur climate data for energy modelling.

Month	**Air Temperature (°C)**	**Relative Humidity (%)**	**Precipitation (mm)**	**Daily solar radiation (kWh/m**^**2**^**/d)**	**Atmospheric pressure (kPa)**	**Wind speed (m/s)**	**Earth temperature (°C)**
**January**	25.8	84.5	219.79	4.63	100.85	2.7	26.64
**February**	26.4	82.0	108.92	5.10	100.82	2.6	27.04
**March**	26.8	83.0	188.79	4.97	100.78	1.9	27.81
**April**	27.2	83.8	186.30	4.62	100.70	1.2	28.55
**May**	27.5	83.3	184.76	4.35	100.65	1.2	28.67
**June**	27.4	81.8	145.80	4.30	100.70	1.5	28.32
**July**	27.1	82.0	167.40	4.35	100.72	1.6	27.85
**August**	27.0	82.0	156.55	4.42	100.74	1.7	27.71
**September**	26.8	82.6	166.80	4.52	100.77	1.5	27.78
**October**	26.8	82.9	187.24	4.34	100.77	1.3	28.01
**November**	27.0	85.5	269.10	3.91	100.77	1.3	27.71
**December**	26.3	86.5	287.99	3.90	100.81	2.0	26.95
**Annual**	25.7	83.3	2269.44	4.45	100.76	1.7	27.76

### 3.2 Energy and emission modelling

The energy modelling in this research was conducted utilizing RETScreen software, which enables the modeler to estimate the energy production and annual emission reduction of a potential project. Equation (1) was used to calculate the annual emission reduction for the application of biomass power plants to reduce diesel power plant usage. The overview of base case emission data or diesel-based power plant data gathered from RETScreen's database that will be used in the calculation is shown in Table 5.

(1) ΔGHG=ABCE−APCE

where *ΔGHG* is the annual emission reduction, *ABCE* is the annual base case emission*,* and *APCE* is the annual proposed case emission.

**Table 5:** Base-case electricity system emission data.

Fuel type	**GHG emission factor excl. T&D (tCO**_**2**_**/MWh)**	**T&D losses (%**)	**GHG emission factor (tCO**_**2**_**/MWh)**
**Diesel**	0.681	9	0.833
**Biomass**	0.027	9	0.126

Other than the biomass power plant, Kundur's electricity is also provided by diesel power plants. To assess the viability of the biomass power plant from the PLN perspective, this research also conducts an energy modelling of diesel power plants which will be used to calculate the cost-savings incurred by the addition of the biomass power plant capacity. The specification of the diesel power plant in this study is shown in Table 7.

**Table 6:** Biomass power plant input data.

**Engine capacity**	400 kW
**Number of engines**	6 units
**Power capacity**	2400 kW
**Heat rate**	18,000 Btu/kWh
**Heat recovery efficiency**	0 %
**Fuel cost**	$13.5/t
**Fuel cost escalation rate**	2 %

**Table 7:** Diesel power plant input data.

**Power Capacity**	2400 kW
**Minimum capacity**	0 %
**Availability**	90 %
**Heat rate**	9200 Btu/kWh
**Heating capacity**	0 %
**Fuel type**	HSD
**Fuel cost**	$0.506/liter

The biomass power plant specification used as the energy modelling input is presented in Table 6.

To provide insights about PLN's de-dieselization program, this study incorporates PLN's cost savings from de-dieselization. Equation (2) was used to calculate PLN's cost savings.

(2) ΔCost=DEPC−BEPC

where *ΔCost* is the cost savings, *DEPC* is the diesel power plant energy production cost and *BEPC* is the biomass power plant energy production cost.

### 3.3 Financial modelling

Financial calculation in this study was conducted using RETScreen software. Several important financial parameters in this research, such as Net Present Value (NPV), Energy Production Cost (EPC) also referred to as Levelized Cost of Electricity (LCOE), Simple Payback (SP), Equity Payback (EP), Internal Rate of Return (IRR), and Benefit-Cost ratio (B-C). NPV was calculated using Equation (3), LCOE was calculated using Equation (4), SP was calculated using Equation (5), EP was calculated using Equation (6), IRR was calculated using Equation (7) B-C ratio was calculated using Equation (8).

(3) $$\mathrm{NPV}=\sum_{n=0}^{N}\frac{Cn}{{\left(1+r\right)}^{n}}$$

(4) $$\mathrm{EPC}=\frac{sum\;of\;cost\;over\;lifetime}{sum\;of\;electricity\;generated\;over\;lifetime}$$

(5) $$\mathrm{SP}=\frac{C-IG}{\left(Cener+Capa+CRE+CGHG\right)-\left(CO\&M+Cfuel\right)}$$

(6) $$\mathrm{EP}=EP=\sum_{n=0}^{N}Cn$$

(7) $$\mathrm{IRR}=0=\sum_{n=0}^{N}\frac{Cn}{{\left(1+IRR\right)}^{n}}$$

(8) $$B-C=\frac{NPV+1\left(1-fd\right)c}{\left(1-fd\right)c}$$

where *N* represents the operating lifespan, *Cn* represents the cash flow in year *n*, *r* represents the discount rate, *C* refers to the initial cost incurred, and *IG* represents incentives and grants. *Cener* stands for the income generated from energy savings, *Ccapa* is the income from capacity savings, *CRE* is the income from renewable energy credit, *CGHG* is the income from GHG reduction, *CO&M* is the operation and maintenance cost, *Cfuel* is the cost of feedstock, and *fd* represents the debt ratio.

The financial input data used in this research is shown in Table 8. The inflation rate was determined by utilizing the average inflation as reported in the most recent Karimun Regency inflation data [48]. Other financial input data were gathered from the RETScreen software database, PLN's electricity supply business plan (RUPTL) [25], IPP's internal document [49], PLN statistics [22], and previous biomass power plant studies.

**Table 8:** Financial input data.

Initial costs	$3050/kW
**O&M costs**	$250/kW
**Discount rate**	12 %
**Inflation rate**	3.12 %
**Project life**	25 years
**Debt ratio**	70 %
**Debt term**	15 years
**Debt interest rate**	10 %

### 3.4 Sensitivity modeling

This research employs sensitivity analysis conducted utilizing RETScreen software to understand the implications of each parameter to the project's financial viability. IPP scenario and IPP expansion scenario were developed to assess the viability of the biomass power plant based on the IPP perspective by utilizing Indonesia's biomass power plant initial tariffs and expansion tariffs. An overview of several parameters included in the IPP scenario and IPP expansion scenario sensitivity analysis is shown in Table 9.

**Table 9:** Input data for sensitivity analysis.

Parameter	Unit	Value	Range (%)	Min.	Max.
**Initial cost**	$/kW	3050	25	2287.5	3182.5
**Electricity exported to grid**	MWh/annum	18,922	25	14,191.5	23,652.5
**Discount rate**	%	12	25	9	15
**Electricity tariff IPP scenario** **Electricity tariff IPP Expansion scenario**	$/MWh $/MWh	110.80 95.90	25 25	138.50 119.88	83.10 71.93
**Feedstock cost**	$/t	13.5	25	10.125	16.875
**Inflation**	%	3.12	25	2.34	3.90
**Operation and maintenance cost**	$/kW	250	25	187.50	312.50
**Debt interest rate**	%	10	25	7.50	12.50

## 4 Results and discussion

### 4.1 IPP scenario

#### 4.1.1 Financial analysis

**Fig. 5:**
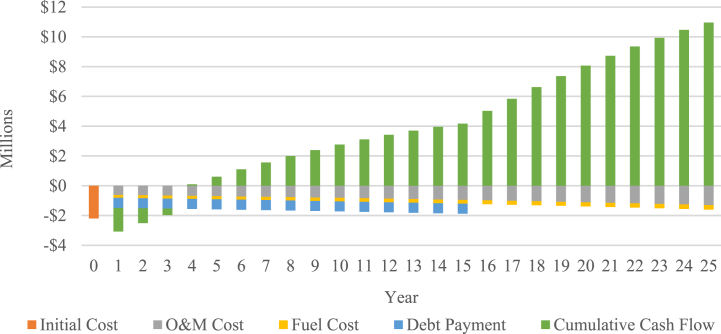
Cash flow of IPP scenario.

A financial analysis was conducted to assess the adequacy of the newly implemented biomass power plant tariff based on the IPP perspective. Electricity exported to grid is calculated at 18,922 MWh/annum, and electricity tariffs at $0.1108/kWh. As shown in Fig. 5, the equity payback period of the IPP scenario occurred after year 3, specifically after 3.8 years. In this case, investors are anticipated to recover their initial equity within a relatively short timeframe, highlighting the tariff sufficiency in generating favorable returns. The amount of time needed to recoup the initial investment, also known as simple payback period, is calculated at 5.6 years. The IPP scenario is projected to generate cumulative cash flows of $10,956,329, NPV of $1,815,146, B-C ratio of 1.8, and IRR of 23.9 %. Based on all the aforementioned aspects, it is considered that the tariff is sufficient to make the project financially viable.

#### 4.1.2 Sensitivity analysis

**Fig. 6:**
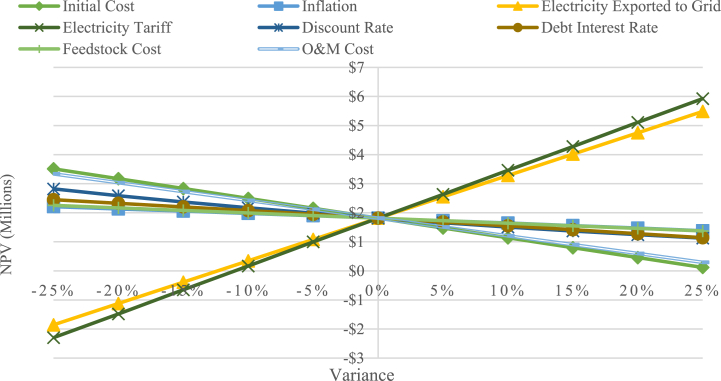
Sensitivity diagram of IPP scenario.

As shown in Fig. 6, the most important variable is the electricity tariff followed by electricity exported to grid. This result is also aligned with [15,18] who reported that the NPV is significantly impacted by electricity tariff and the amount of electricity exported to the grid. The next most important parameter is initial cost, where a 25 % initial cost increase would decrease the NPV to $119,074. The least critical parameter is inflation, where an increase of 25 % results in a 23.9 % NPV decrease.

### 4.2 IPP expansion scenario

#### 4.2.1 Financial analysis

**Fig. 7:**
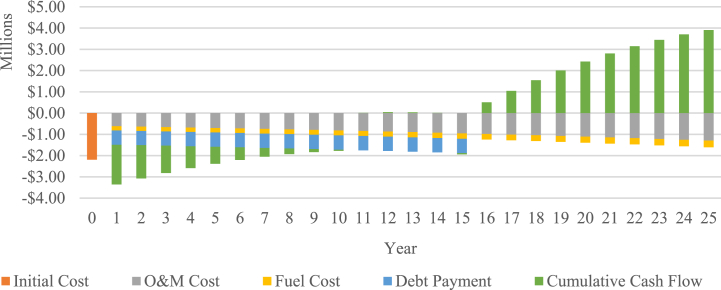
Cash flow of IPP Expansion scenario.

Electricity exported to grid is calculated at 18,922 MWh/annum, with a corresponding electricity tariff of $0.0959/kWh. As shown in Fig. 7, by the end of the project's lifetime the cumulative cash flows are calculated at $3,908,029, corresponding to an NPV of -$396,085, and B-C ratio of 0.82, and an IRR of 9.3 %. The negative NPV result, B-C ratio lower than 1, and IRR value of less than the discount rate collectively conclude that the newly implemented biomass expansion tariff is insufficient to make the project financially viable.

#### 4.2.2 Sensitivity analysis

**Fig. 8:**
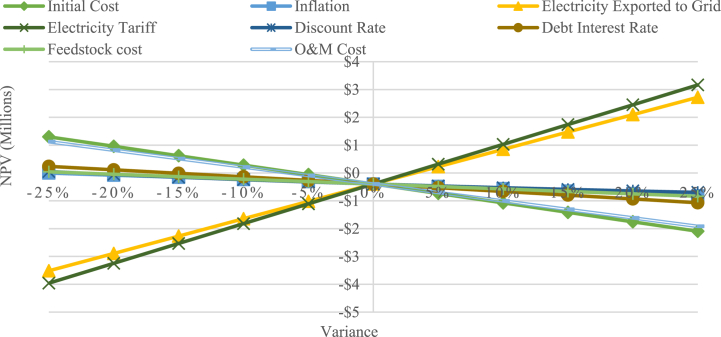
Sensitivity diagram of IPP expansion scenario.

As shown in Fig. 8, the newly implemented biomass expansion power plant tariff is inadequate to make the project financially feasible, as indicated by the negative NPV result. To achieve financial viability based on the expansion tariff, one of these factors needed to be adjusted. The electricity exported to grid needs to be higher by 5 %, equivalent to a capacity factor of 94.5 %. Nevertheless, attaining this goal is exceptionally challenging due to factors such as downtime for maintenance, variations of feedstock quality and availability, and losses in electricity. To achieve financial viability within tariff limitation, one of these factors needs to be adjusted. Either initial cost by 10 %, discount rate by 25 %, debt interest rate by 20 %, feedstock cost by 25 %, or O&M cost by 10 %. It is important to mention, that even a 25 % decrease in inflation is insufficient to make the project financially viable. However, based on the scenario, a 5 % increase in electricity tariff is needed to make the project feasible.

### 4.3 PLN scenario

#### 4.3.1 Cost of energy analysis

**Fig. 9:**
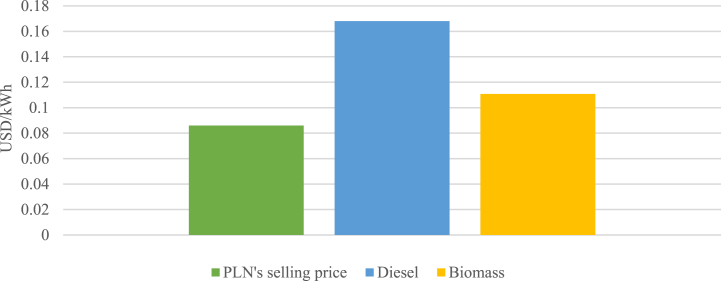
Cost of energy comparison.

Since PLN's selling tariffs are regulated by the government, the cost of energy becomes a crucial factor in maintaining financial viability and energy accessibility. To achieve this, minimizing the cost of energy becomes imperative for the company. The cost of energy comparison of a biomass power plant and a diesel power plant is shown in Fig. 9. The figure highlights that the cost of obtaining energy from the 2.4 MW biomass power plant of $0.1108/kWh, making the biomass power plant a more cost-effective option in comparison to the diesel power plant with $0.1680/kWh, resulting in a cost savings value of $0.0572/kWh.

**Fig. 10:**
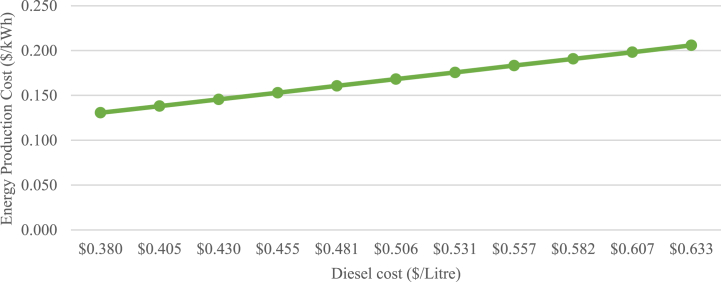
Sensitivity diagram of diesel EPC.

The PLN scenario was developed to assess the effectiveness of PLN's de-dieselization program. A sensitivity diagram of energy production cost with the diesel power plant is presented in Fig. 10.

#### 4.3.2 Financial analysis

**Fig. 11:**
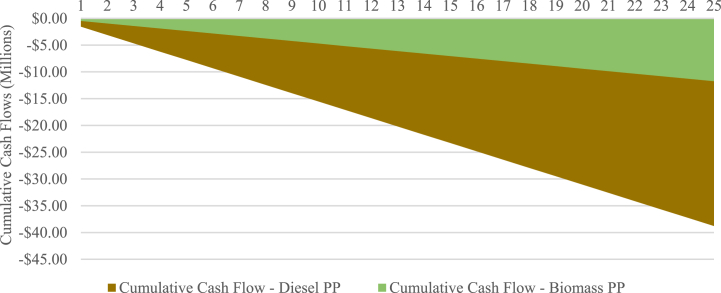
Cumulative cash flows of PLN scenario.

To further analyze the financial viability of the PLN scenario, a cumulative cash flow projection is presented in Fig. 11. As shown in the figure, the cumulative cash flow of the diesel power plant is -$38,789,280, whereas the biomass power plant stands at -$ 11,731,392. From these findings, cumulative cost savings of $27,057,888 throughout the project's lifespan are identified, corresponding to an NPV of $8,488,751 and a B-C ratio of 2.31. Based on those results, the introduction of the biomass power plant with a PPA tariff of $0.1108 is considered financially feasible for PLN.

#### 4.3.3 Sensitivity analysis

**Fig. 12:**
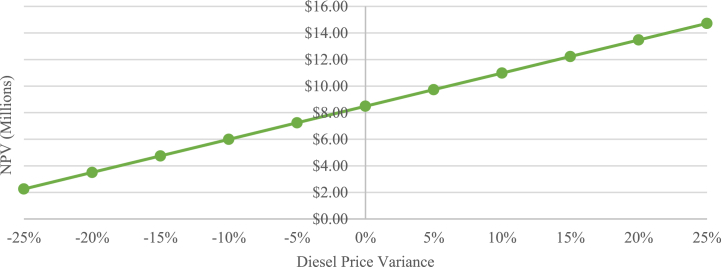
Sensitivity diagram of PLN scenario NPV.

To further analyze the adequacy of the tariff to the financial viability of the PLN scenario, a sensitivity analysis is conducted, as presented in Fig. 12. The results showed that the NPV of the PLN scenario is significantly impacted by the diesel price. According to the calculation, even with a 25 % decrease in diesel price, the project is still financially feasible, due to its high NPV results resulting from de-dieselization.

### 4.4 Scenario comparison

**Fig. 13:**
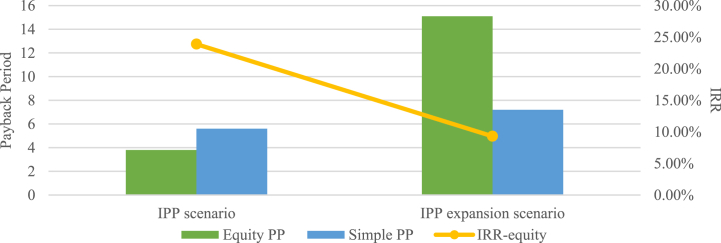
Payback period and IRR comparison.

The payback period and IRR comparison of the two scenarios are presented in Fig. 13. It is shown that the IPP expansion scenario would increase the equity payback period and simple payback period to 15.1 years, and 7.2 years respectively. Conversely, the IRR-equity of the expansion scenario decreased from 23.89 % to 9.30 %.

**Fig. 14:**
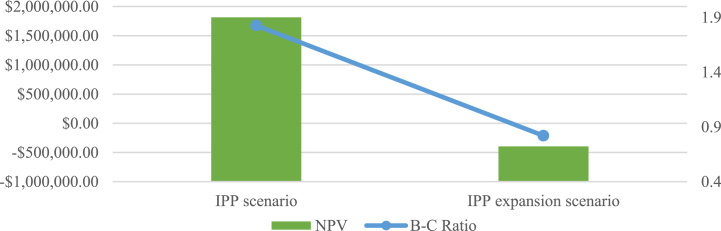
NPV and B-C ratio comparison.

The NPV and B-C ratio comparison of the two scenarios is presented in Fig. 14, indicating the profitability of the project based on the two scenarios. The data mentioned above suggests that the project would be financially feasible based on the newly implemented biomass tariff, however, based on the newly implemented biomass expansion tariff, the project would be financially unfeasible.

**Fig. 15:**
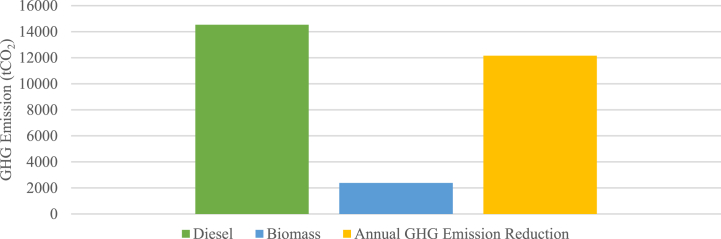
Annual GHG emission comparison.

An annual GHG emission comparison is shown in Fig. 15, indicating that the biomass power plant achieves lower GHG emissions compared to the diesel power plant, with a value of 14,536 tCO_2_ and 2381 tCO_2_ respectively, resulting in a GHG emission reduction of 12,155 tCO_2_ annually.

### 4.5 Discussion

A comparison of the research results was made to analyze the study's results further compared with those of other studies. As shown in Table 10, the results demonstrate that Indonesia's biomass tariffs, set at $0.1108/kWh for the initial phase and $0.0959/kWh for the expansion phase, are substantially lower compared to countries such as Portugal, Fiji, and India. Specifically, Portugal's biomass project has a tariff of $0.121/kWh, Fiji has the highest at $0.162/kWh, and India's biomass gasification projects in microgrid regions are at $0.156/kWh. These disparities strongly suggest that Indonesia's current tariff framework is inadequate to ensure the financial viability and long-term sustainability of its biomass sector. To foster increased investment, particularly within the de-dieselization initiative aimed at remote and microgrid regions, it is recommended that Indonesia's tariffs be revised to incorporate regional average generation costs. Such adjustments would enhance the economic attractiveness of biomass projects, reduce dependence on diesel-based power generation, and contribute to lowering the overall cost of electricity. This would ultimately support greater energy accessibility and affordability while promoting a more sustainable energy mix.

**Table 10:** Results comparisons.

Author	Scenario	Year	Technology	Region	Capacity (MW)	Tariffs ($/kWh)	NPV (Million $)	Annual power production (GWh)	EPC/LCOE (USD/kWh)
This research	IPP Initial	2024	Gasification	Indonesia	2.4	0.1108	1.8	18.922	0.098
	IPP Expansion	2024	Gasification	Indonesia	2.4	0.0959	−0.396	18.922	0.098
[18]^†^		2018	Gasification	Portugal	11	0.121	2.6	78.436	
[15]		2021	Steam turbine	Fiji	10	0.162	16.1	74.816	0.140
[17]^‡^		2020	Gasification	India	0.048				0.156

† Currency exchanged from EUR to USD – 1 USD = 0.9 EUR.

‡ Currency exchanged from INR to USD – 1 USD = 80 INR.

## 5 Conclusion

Indonesia is committed to achieving a 23 % renewable energy mix by 2025 and net zero emissions by 2060. However, the adequacy of tariffs creates challenges for the biomass power sector. Financial analysis shows that the initial tariff is sufficient for attaining financial viability, while expansion tariffs are inadequate, requiring a capacity factor exceeding 94.5 %, which is difficult to attain due to operational and maintenance. Sensitivity analysis shows that electricity tariff and electricity export quantity are two of the most important parameters in ensuring the biomass project's economic sustainability.

Transitioning from diesel to biomass can significantly lower energy production costs, with Kundur's projected biomass power plant having an EPC of $0.099/kWh compared to diesel's $0.168/kWh. Emission analysis highlights an 83.7 % decrease in emissions emitted with the utilization of BPP compared to diesel power plants, supporting both economic and environmental sustainability. To achieve energy transition, it is imperative to incorporate average generation cost in formulating future renewable energy tariffs to ensure economic sustainability for both PLN and IPPs, stimulate renewable energy utilization, and make energy more accessible for everyone.

To supplement this research, there are several suggestions for further research. A more technical exploration of the biomass conversion process is suggested, including a comparison of various biomass types to identify the most cost-effective feedstocks. Additionally, an extension of the study to encompass alternative technology and other locations is also suggested, aiming to provide a more comprehensive understanding of Indonesia's renewable energy tariffs. Nevertheless, one does expect that the findings of this research hold in other locations. That a specific renewable energy technology does matter in lowering energy generation costs thus making energy more accessible for everyone.

## Funding statement

This research did not receive any specific grant from funding agencies in the public, commercial, or not-for-profit sectors.

## Data availability statement

Data included in article/supp. material/referenced in article.

## CRediT authorship contribution statement

**Indra A. Aditya:** Supervision, Resources, Investigation, Data curation. **Hendry Timotiyas Paradongan:** Writing – review & editing, Writing – original draft, Methodology, Investigation, Formal analysis. **Iswan Prahastono:** Supervision, Resources, Data curation. **Sudjono Kosasih:** Supervision, Investigation, Data curation. **Kevin M. Banjar-Nahor:** Supervision, Investigation, Formal analysis. **Ngapuli Irmea Sinisuka:** Supervision, Investigation, Data curation, Conceptualization.

## Declaration of competing interest

The authors declare that they have no known competing financial interests or personal relationships that could have appeared to influence the work reported in this paper.
